# Consultation on kidney stones, Copenhagen 2019: lithotripsy in percutaneous nephrolithotomy

**DOI:** 10.1007/s00345-020-03383-w

**Published:** 2020-07-29

**Authors:** Tomas Andri Axelsson, Cecilia Cracco, Mahesh Desai, Mudhar Nazar Hasan, Thomas Knoll, Emanuele Montanari, Daniel Pérez-Fentes, Michael Straub, Kay Thomas, James C. Williams, Marianne Brehmer, Palle J. S. Osther

**Affiliations:** 1 Division of Urology, Department of Clinical Sciences, Danderyd Hospital, Karolinska Institute, Solna, Sweden; 2 Department of Urology, Cottolengo Hospital of Torino, Turin, Italy; 3 Muljibhai Patel Urological Hospital, Nadiad, Gujarat India; 4 Department of Urology, Klinikum Sindelfingen-Boeblingen, University of Tübingen, Sindelfingen, Germany; 5 Urological Dept. at Fondazione Ca Granda-Ospedale Maggiore Policlinico of Milan, University of Milan, Milan, Italy; 6 Department of Urology, University Hospital of Santiago de Compostela, Santiago de Compostela, Spain; 7 Department of Urology, University Hospital Klinikum rechts der Isar, Technical University Munich, Munich, Germany; 8 Stone Unit, Guy’s and St Thomas’ NHS Foundation Trust, London, UK; 9 Department of Anatomy, Cell Biology and Physiology, Indiana University School of Medicine, Indianapolis, IN USA; 10 Urological Research Center, Department of Urology, Lillebaelt Hospital, University of Southern Denmark, Vejle, Denmark

**Keywords:** Kidney calculi, PCNL, Lithotripsy, Ballistic, Ultrasonic, Laser

## Abstract

**Purpose:**

To evaluate the balance between existing evidence and expert opinions on the safety and efficacy of new technological improvements in lithotripsy techniques for percutaneous nephrolithotomy (PCNL).

**Methods:**

A scoping review approach was applied to search literature in Pubmed, Embase, and Web of Science. Consensus by key opinion leaders was reached at a 2-day meeting entitled “Consultation on Kidney Stones: Aspects of Intracorporeal Lithotripsy” held in Copenhagen, Denmark, in September 2019.

**Results:**

New-generation dual-mode single-probe lithotripsy devices have shown favourable results compared with use of ballistic or ultrasonic lithotripters only. However, ballistic and ultrasonic lithotripters are also highly effective and safe and have been the backbone of PCNL for many years. Compared with standard PCNL, it seems that mini PCNL is associated with fewer bleeding complications and shorter hospital admissions, but also with longer operating room (OR) time and higher intrarenal pressure. Use of laser lithotripsy combined with suction in mini PCNL is a promising alternative that may improve such PCNL by shortening OR times. Furthermore, supine PCNL is a good alternative, especially in cases with complex renal stones and large proximal ureteric stones; in addition, it facilitates endoscopic combined intrarenal surgery (ECIRS).

**Conclusion:**

Recent technological improvements in PCNL techniques are promising, but there is a lack of high-level evidence on safety and efficacy. Different techniques suit different types of stones and patients. The evolution of diverse methods has given urologists the possibility of a personalized stone approach, in other words, the right approach for the right patient.

## Introduction

Percutaneous nephrolithotomy (PCNL) was first described in 1976 by Fernström and Johansson as an operative technique for the removal of kidney stones through a percutaneous nephrostomy tract [1]. The approach was further developed in subsequent years, as illustrated by a case series published by Alken in 1981, which showed promising results in treatment of stones when using direct percutaneous ultrasound lithotripsy and stone extraction [2]. Further improvements in PCNL over the last decades have led to this method becoming one of the cornerstones in the treatment of large kidney stones, alongside shock wave lithotripsy (SWL) and retrograde intrarenal surgery (RIRS). In the EAU guidelines, PCNL is the standard procedure for large renal calculi (> 2 cm). The choice of endoscope, lithotripsy technique, and access tract size is made at the discretion of the surgeon and is not standardized [3]. The evolution of smaller tract sizes with the mini PCNL procedure and the effect of that approach on complication rates and stone-free rates (SFRs) are still not clear, and there is a scarcity of randomized controlled trials and high-quality research in this area.

To evaluate the balance between existing evidence, expert opinions, and the safety and efficacy of new technological improvements in lithotripsy in PCNL, key opinion leaders in the field were invited to evaluate and discuss the available evidence at a 2-day meeting entitled “Consultation on Kidney Stones: Aspects of Intracorporeal Lithotripsy “held in Copenhagen, Denmark, in September 2019. The experts were assigned different topics and prepared their presentations through scoping reviews achieved by scanning the literature using PubMed, EMBASE, and Web of Science. The first day of the meeting was open only to the experts, who first presented their topics for each other, after which the presentations were discussed within the group and were subsequently adjusted if necessary. The second day was open to a global audience, with all the experts giving their presentations, and this was followed by free discussions.

## PCNL techniques

Conventional PCNL requires an access tract of 24–30F, and lithotripsy is traditionally performed using a rigid probe. The fragmentation device is usually ballistic, ultrasonic, or combined, although laser fragmentation has recently gained in popularity. Compared with the laser technique, ballistic and ultrasonic disintegration have the disadvantage of requiring straight access to the stone. Mini PCNL is usually classified as using tract sizes of 14–20F, and lithotripsy is generally performed by laser disintegration although other methods are possible [4]. Desai et al. described ultra-mini PCNL with an access tract of 11–13F using a 6F nephroscope; with this method only laser can be used for lithotripsy [4, 5]. Micro PCNL as reported by Desai in 2011 uses a 4.85F all-seeing needle with a 16-gauge needle sheath and a three-way connector that allows irrigation and passage of a flexible telescope and a 200-μm Holmium laser fibre [6].

### Technical and safety aspects of ballistic, ultrasonic, and combined lithotripsy

#### Ballistic lithotripsy

Ballistic lithotripters use compressed air to accelerate a projectile inside the handpiece, which then hits the probe to elicit a shock wave that moves through the probe to the stone to cause mechanical fragmentation. This leads to significant retropulsion of the stone, and therefore, the stone must be pushed against the wall of the collecting system to optimize fragmentation and avoid loss of fragments. In most cases, both pressure and frequency can be altered on the device. The safety benefits of this technique are mainly associated with the absence of heat generation during lithotripsy, which means that there is no risk of thermal injury.

The Swiss Lithoclast^®^ (EMS) emerged in 1991 as the first available percutaneous ballistic lithotripsy device. A case series of 145 ureteroscopy (URS) procedures conducted by Yinghao et al. in 2000 resulted in stone-free rates (SFRs) of 55% after 1 month and 78% after 2 months [7]. All those procedures were performed with a rigid ureteroscope (9.5 or 10.5F). In addition, secondary procedures such as SWL were common, and 3.4% had perforations of the ureter, the majority of which could be handled conservatively with stenting. The Cook LMA™ StoneBreaker™ is a ballistic lithotripter that offers the benefit of being portable, because it has self-contained CO_2_ pressure cartridges. Each cartridge can deliver 80–100 shocks, and there is no need for an external source of compressed air. In 2008, Nerli et al. published a prospective study of 110 patients undergoing URS with the StoneBreaker™ that showed promising results [8]. In that investigation, the mean stone size was 1.3 cm, and a mean of eight shocks was used for disintegration; no complications were reported. In 2011, Chew et al. reported the results of the Canadian StoneBreaker trial, which was a randomized controlled trial (RCT) comparing use of the LMA StoneBreaker™ and the Swiss Lithoclast^®^ during percutaneous nephrolithotripsy [9]. That assessment showed that the StoneBreaker™ provided faster stone fragmentation and total lithotripsy time, as well as shorter setup time, although there was no difference in SFR between these devices.

#### Ultrasonic lithotripsy

Ultrasonic lithotripters use piezoceramic crystals that convert electrical energy into mechanical energy, creating ultrasonic waves at 23–25 kHz. Acoustic waves from the handpiece cause the tip to vibrate, and fragmentation is achieved when the probe tip comes in contact with the stone. Probes are available from 2.5 to 6.0F. Ultrasonic probes are hollow and require continuous irrigation. The irrigation fluid and stone fragments are suctioned out through the probe, which has a cooling effect on the probe and the handpiece to prevent overheating [10].

An RCT performed by Radfar et al. in 2017 compared the safety and efficacy of ultrasonic versus ballistic lithotripsy in PCNL [11]. No significant difference between the two techniques was found regarding SFRs or complications, but significantly shorter stone clearance time was observed when using ballistic lithotripsy for harder stones and ultrasonic lithotripsy for soft stones. Thus, it seems that these two probes have advantages for different stone types, leading to development of combination probes. Recently, the ultrasonic lithotripsy technology has been further developed to more precisely controlling probe vibration (UreTron). Initial experience with this device was tested in a non-randomized, prospective comparison, and the Urotron lithotripter was found to achieve the highest stone clearance rate (59 mm^2^/min) in comparison to three state-of-art lithotripters (CyberWand™, StoneBreaker™, and Swiss LithoClast Select™) [12].

### Two-probe dual-modality (ballistic and ultrasonic) lithotripsy

Dual-modality lithotripsy combines an ultrasonic device with a ballistic component to exploit the benefits of both devices and thereby improve the overall efficiency and versatility of lithotripsy. The CyberWand™ developed by Olympus is a dual ultrasonic/ballistic device with an inner hollow probe that vibrates at 21,000 Hz and a larger ballistic outer probe that moves at a lower frequency of 10 Hz. The two probes are connected to a single handpiece, and the inner probe can be activated alone or in conjunction with the outer probe. Direct contact with the stone is required for fragmentation.

The Swiss Lithoclast^®^ Master (EMS) is also a combined ultrasonic and ballistic lithotripter. The ballistic probe is positioned as a rod inside the hollow ultrasonic probe. The two modes, ultrasonic and ballistic, can be used individually or in combination. The basic principle of the Swiss Lithoclast^®^ Master is that the ultrasonic probe makes direct contact with the stone, and, when the ballistic probe is activated, the tip projects past the end of the ultrasonic probe and provides additional ballistic fragmentation. When the ballistic probe is retracted back within the ultrasonic probe, the ultrasonic fragmentation continues.

An RCT performed by Lehman et al. compared a combined ultrasonic and ballistic lithotripter (Swiss Lithoclast^®^ Master) with a standard ultrasonic lithotripter in PCNL [13], and the results demonstrated that the dual mode was faster for fragmentation of hard stones but slower for soft stones, and there was no difference in OR time or SFRs. An in vitro study comparing the CyberWand™ and Swiss Lithoclast^®^ Master also showed a faster fragmentation time for the Cyberwand [14]. In a multicentre RCT comparing the CyberWand™ with a single probe ultrasonic lithotripter (Olympus LUS-II), Krambeck et al. found no difference in clinical outcomes, although the malfunction rate was higher with the CyberWand™ [15]. Another RCT conducted by York et al. compared the CyberWand™, Lithoclast Select (combination ballistic and ultrasonic device), and the Cook LMA™ StoneBreaker, and the different devices provided similar adjusted stone clearance rates for stones of > 2 cm and also offered comparable safety and efficacy [16].

### Single-probe dual-modality (ballistic and ultrasonic) lithotripsy

The Olympus ShockPulse™, FDA approved in 2014, is a single-probe (2.91–11.3F) dual-action lithotripsy system that uses constant ultrasonic wave energy with intermittent ballistic shock wave energy. The device is controlled by buttons on the handpiece or by foot pedals, and the larger probes are hollow and equipped with a suction system. An in vitro study by Chew et al. compared the ShockPulse™ with the CyberWand™, Swiss LithoClast^®^ Master, and Olympus LUS-II [17]. The ShockPulse™ was faster than the LUS-II and Swiss LithoClast^®^ Master at both stone fragmentation and evacuation of the fragments. This can probably be explained by the fact that the lumen of ShockPulse™ is larger than the lumens of the two-probe combined devices, which are partly occupied by the ballistic/pneumatic probe. The same applies to the Swiss Lithoclast Trilogy^®^ (EMS), approved in 2018, which has a single-probe (3.3–11.7F) design and uses ultrasonic and electromagnetic energy. This device applies suction through a hollow tube and has a foot pedal to control suction and lithotripsy. An in vitro study published by Carles et al. in 2018 showed that Lithoclast^®^ Trilogy had faster stone clearance time compared with ShockPulse™ and Swiss Lithoclast^®^ Select (Master) [18]. In a recent series comprising 31 cases, Swiss Lithoclast^®^ Trilogy was shown to be highly effective in both standard and mini PCNL, for which mean stone volume clearance ratios were 590.7 and 370.5 mm^3^/min, respectively [19].

In an evaluation of tissue damage induced by different lithotripter devices, Khoder et al. compared Swiss Lithoclast^®^ Trilogy with two other ultrasound lithotripters (Storz Calcuson^®^ and Swiss LithoClast^®^ Vario) [20]. The lithotripter probes were put in direct contact with bladder tissue from pigs for 10 s, and thereafter the histological features were assessed. The authors found no significant differences between the tested devices with regard to histological findings, which indicates that the studied devices offered comparable safety. The handpiece of Swiss Lithoclast^®^ Trilogy is significantly heavier than that of ShockPulse™, which may be important from the perspective of ergonomics. However, the impact of functional design on surgical outcome has not yet been sufficiently evaluated for the two combined lithotripters or for any other lithotripters.

## Comparing clinical outcomes with conventional and miniaturized PCNL in the context of lithotripsy

Lithotripsy devices for miniaturized PCNL consist of lasers, ballistic and dual-modality lithotripters. Traditionally Holmium lasers have been used, applying the so-called Vacuum Cleaner effect for fragment clearance [21]. Due to the fact that the probes of the dual-modality lithotripters have to be down-sized in miniaturized PCNL, there is a risk that fragments will block the hollow probes. Recently, however, the Swiss Lithoclast^®^ Trilogy was found feasible even in mini PCNL due to optimized suction [22]. Although this suggests a role for dual-modality lithotripters in mini PCNL, further evaluation in comparative studies and clinical trials is warranted. In the following lithotripsy data from clinical series in miniaturized PCNL will be discussed.

When comparing clinical outcomes between mini and standard PCNL, we specifically considered lithotripsy-related outcomes such as SFRs, OR time, costs, and need for ancillary treatments. Karakan et al. conducted a randomized trial comparing ultra-mini PCNL (14F) with standard PCNL (26F) and found comparable SFRs for calculi of ≤ 2.5 cm [23]. In that study, lithotripsy was performed by holmium laser in the ultra-mini PCNL group, and ballistic lithotripsy energy was used for standard PCNL. SFR was 88% for standard PCNL compared with 89.3% for the ultra-mini PCNL, indicating similar efficiency for the two methods.

In a randomized study performed by Song et al., mini PCNL (16F) using laser and suction was assessed in comparison with combined ballistic and ultrasonic lithotripsy applied in standard PCNL [24]. The results showed that the mini PCNL technique was more efficient than standard PCNL after one procedure, with SFRs of 89% versus 58%. SFR was evaluated by plain abdominal radiography 3–5 days after surgery. The mini PCNL procedures with suction were also faster than the standard PCNLs. In another investigation, mini PCNL using laser disintegration was compared with standard PCNL using combined ballistic and ultrasonic lithotripsy for stones larger than 2 cm, and both techniques were found to be equally effective, regardless of whether the patients had a single stone or multiple calyceal stones [25].

Focusing specifically on staghorn calculi, Zhong et al. studied 54 patients who had such calculi and were randomized to either mini PCNL or standard PCNL [26]. The data obtained showed that mini PCNL with multiple tracts was more effective than a standard PCNL with a single tract. In short, the mini PCNL had a higher SFR of 89.7% (compared with 68% for standard PCNL) and was associated with fewer ancillary treatments.

In a meta-analysis including eight trials with a total of 749 patients, mini PCNL was compared with standard PCNL, and the authors found no difference in SFRs [27]. However, the mini PCNL patients had shorter hospital stays and fewer blood transfusions than the patients in the standard group. On the other hand, OR time was longer for mini PCNL than for standard PCNL.

The efficacy of mini PCNL for larger stones has been debated. Kokov et al. reported the SFR to be 42.5% after mini PCNL, where stone size was the only independent risk factor for residual fragments [28]. In a systematic review on the efficacy and safety of mini PCNL published by the European Association of Urology Urolithiasis Guidelines Panel in 2017, it was concluded that available studies do suggest that mini PCNL is at least as efficient as standard PCNL [29]. However, only two RCTs were included in that review, and those trials differed with regard to the tract sizes used and the type of stones treated [30, 31]. In another RCT, Ganesamoni et al. compared ballistic lithotripsy with laser lithotripsy in mini PCNL and found that the former approach created larger fragments and more stone migration, and also required more stone retrieval, whereas the two approaches did not differ with respect to fragmentation time and SFR [32].

**Fig. 1:**
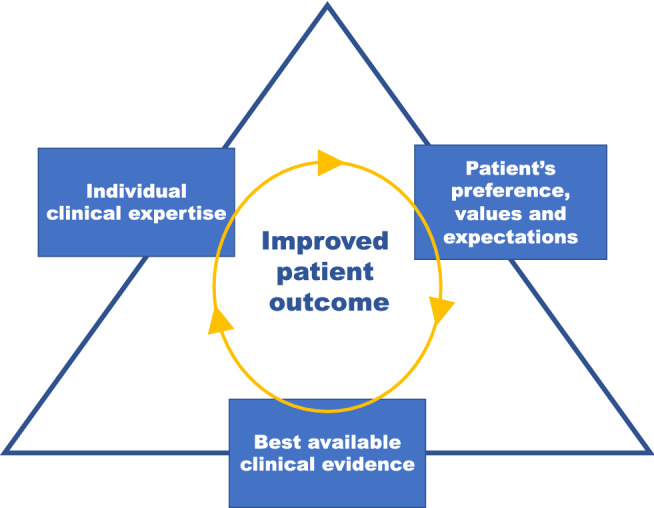
Approach to individualized stone treatment

Overall, there are severe problems when trying to draw conclusions about clinical outcomes of PCNL from different studies. On the one hand, there is a lack of high-quality RCTs, and retrospective studies are often flawed by selection bias. On the other hand, the heterogenous nature of stone disease makes it very difficult to perform meaningful RCTs, because such studies typically include very selective cases that do not necessarily reflect daily clinical practice. Furthermore, surgeons have their preferences and so do patients, which means that it is not always possible to translate the results of RCTs into local clinical practice. In addition, differences in the techniques used, tract size, stone size and complexity, definitions of SFR, modality of defining SFR (US/KUB/CT) and the time point at which SFR is evaluated are often not reported in a uniform manner, which makes comparisons problematic. From this perspective, evidence-based medicine (EBM) with regard to aspects of lithotripsy in PCNL will to a large extent have to rely on clinical expertise and patient’s preference, values, and expectations rather than on high-level scientific evidence (Fig. 1). With a lack of high-level evidence supporting different lithotripsy techniques, it is especially important to consider safety aspects of newer approaches such as miniaturized PCNL.

### Safety aspects of lithotripsy during miniaturized PCNL

To perform PCNL safely, it is necessary to have appropriate endoscopic vision, cooling of the lithotripsy environment to prevent overheating, and sufficient expansion of the collecting system for atraumatic movements. There is also a need for a safe way to evacuate stone fragments effectively in mini PCNL. Maintaining control over intrarenal pressure is important as well, because there is evidence that high intrarenal pressure during PCNL increases the risk of postoperative morbidity [33, 34]. Furthermore, a rise in such pressure may cause reduced renal blood flow, pyelovenous backflow, and pyelolymphatic backflow, which may potentially cause infectious and bleeding-related complications [34]. In addition, increase in intrarenal pressure leads to extravasation of fluid, potentially leading to postoperative pain. Clearly, the risks and morbidity of intrarenal reflux, pelvic perforation, thermal injury, and serious infectious and bleeding complications need to be thoroughly evaluated and compared with standard PCNL.

There are concerns that mini PCNL may cause higher intrarenal pressures due to less efficient drainage of irrigation fluid. A study by Tepeler et al. found significantly higher intrarenal pressure in mini PCNL procedures than in standard PCNLs, although outcomes in both were similar in terms of complications and stone clearance [31]. Omar et al. evaluated the impact of irrigation pressures in standard PCNL on risk of systemic inflammatory response syndrome (SIRS) and observed that high-pressure fluid irrigation was a risk factor for postoperative SIRS [35]. Chu et al. focused specifically on the differences between standard and mini PCNL and found comparatively higher intrarenal pressures for mini PCNL, and also noted that mini PCNL procedures were more likely to be associated with postoperative sepsis [36]. In a porcine model, mini PCNL was associated with higher intrarenal pressures and higher risk of organ bacterial seeding in the setting of an infected collecting system, suggesting a higher potential for infectious complications [37]. Suction through the sheath or other intrarenal pressure-regulating systems (e.g., new designs of the sheath) may overcome the concerns regarding pressure in mini PCNL, and suction through the sheath during miniaturized PCNL definitely deserves attention with respect to both efficacy and safety [38–40].

### Lithotripsy and stone clearance in supine PCNL

There are no guidelines for the use of supine or prone PCNL, and the existing evidence in this area is limited primarily to sparse expert opinions. In supine PCNL, the nephroscope is inserted from below through a horizontal or slightly downward inclined Amplatz sheath. This angle uses gravity to drain the irrigation fluid and the stone fragments. Accordingly, the collecting system is less distended, and the intrarenal pressure may be lower than in the prone position, which may cause reduced vision and working space but potentially reduces the risk of postoperative infection [41, 42]. Despite the plausibly lower intrarenal pressure in supine PCNL, it is possible that fragments can descend into the ureter more easily than in the prone position due to gravitational effects. However, in endoscopic combined intrarenal surgery (ECIRS) this is not a problem, because a flexible ureteroscope is positioned in the ureter, both to occupy that position and to detect fragments that might drain into the ureter [43, 44].

ECIRS has several benefits, especially for larger stones that might otherwise require multiple access tracts or a two-step procedure to attain complete stone fragmentation. Using only one percutaneous access tract in a one-step PCNL, in combination with flexible ureteroscopy, it is possible to minimize morbidity without sacrificing the quality of stone fragmentation or clearance of complex stones [45]. The flexible ureteroscope contributes in a multitude of ways: it has a preliminary diagnostic and intraoperative role helping in real-time procedural choices; it can reach calyces that are difficult or even impossible to reach with a rigid nephroscope; it actively contributes to achieving endoscopic vision and to stone fragmentation and reduces radiation exposure; it is also helpful at the end of the operation to evaluate the calyceal system for residual fragments [46]. An additional benefit of ECIRS is that a stone in a calyx awkward for RIRS may be passed to the nephroscope for stone fragmentation (‘passing the ball’).

ECIRS in the Galdakao-modified supine Valdivia (GMSV) position has also been shown to offer good results in the treatment of impacted proximal ureteral stones [43]. The use of a combined approach creates an open, low-pressure system that reduces the absorption of irrigation fluid into the circulation [47]. This makes it possible to push the stone/stone fragments up into the renal pelvis and subsequently remove them via the percutaneous access. This in turn reduces the risk of ureteral injury that is associated with lithotripsy and stone basketing.

In a meta-analysis of 15 RCTs with a total of 1474 patients, Lie et al. compared supine versus prone position in PCNL and found that operative time in supine PCNL was shorter [48]. As also mentioned above, Lie and colleagues noted that the downward or horizontal angle of the Amplatz sheath facilitated faster evacuation of stone fragments with the help of gravity. Furthermore, these investigators found lower rates of postoperative fever in the supine group than in the prone group, which confirms data from the large CROES PCNL study [49].

## Conclusion

Today, PCNL is not standardized in terms of technical considerations such as access tract size and devices used for stone disintegration. Standard PCNL (24–30F) enables the use of probes that make it possible for fragments to be both disintegrated and removed by suction through the same probe. Older devices that rely purely on ballistic or ultrasonic techniques are still viable and have proven their efficacy and safety over many years. New technological advances including single-probe dual-modality lithotripters using a combination of ultrasonic and ballistic techniques are promising, and seem to be more effective than single-energy probes. Mini PCNL requires smaller probes for lithotripsy, and larger fragments cannot be extracted through the probe. However, small fragments resulting from laser lithotripsy can be effectively evacuated using the vacuum cleaner effect [21, 50]. New developments such as the use of laser lithotripsy in combination with suction devices in mini PCNL appear to be safe and effective, and may even further increase the efficacy and safety of mini PCNL. Moreover, laser probes are flexible and can be used in flexible endoscopic devices. There is limited quality evidence comparing standard and mini PCNL, although research has indicated that mini PCNL is associated with fewer bleeding complications and shorter hospital admissions [27]. However, a drawback of mini PCNL is that it apparently requires longer OR time [27]. On the other hand, the use of laser lithotripsy combined with suction may be a promising alternative that can potentially improve mini PCNL performance by shortening OR times [23, 24].

In that context, using supine positioning of patients during PCNL is a good alternative, especially in complex renal stone situations that require a combined approach (i.e., ECIRS). However, there is no evidence that one method is better than the other, such as standard PCNL with ballistic and/or ultrasonic disintegration or mini PCNL with laser disintegration. However, the evolution of different techniques has increased the possibilities of a personalized approach, in other words, the right method for the right patient.
