# Correction to: Consultation on kidney stones, Copenhagen 2019: lithotripsy in percutaneous nephrolithotomy

**DOI:** 10.1007/s00345-020-03565-6

**Published:** 2020-12-30

**Authors:** Tomas Andri Axelsson, Cecilia Cracco, Mahesh Desai, Mudhar Nazar Hasan, Thomas Knoll, Emanuele Montanari, Daniel Pérez-Fentes, Michael Straub, Kay Thomas, James C. Williams, Marianne Brehmer, Palle J. S. Osther

**Affiliations:** 1 Division of Urology, Department of Clinical Sciences, Danderyd Hospital, Karolinska Institute, Solna, Sweden; 2 Department of Urology, Cottolengo Hospital of Torino, Turin, Italy; 3 Muljibhai Patel Urological Hospital, Nadiad, Gujarat India; 4 Department of Urology, Klinikum Sindelfingen-Boeblingen, University of Tübingen, Sindelfingen, Germany; 5 Urological Dept. at Fondazione Ca Granda-Ospedale Maggiore Policlinico of Milan, University of Milan, Milan, Italy; 6 Department of Urology, University Hospital of Santiago de Compostela, Santiago de Compostela, Spain; 7 Department of Urology, University Hospital Klinikum rechts der Isar, Technical University Munich, Munich, Germany; 8 Stone Unit, Guy’s and St Thomas’ NHS Foundation Trust, London, UK; 9 Department of Anatomy, Cell Biology and Physiology, Indiana University School of Medicine, Indianapolis, IN USA; 10 Urological Research Center, Department of Urology, Lillebaelt Hospital, University of Southern Denmark, Vejle, Denmark

## Correction to: World Journal of Urology 10.1007/s00345-020-03383-w

In the original publication of the article, we inadvertently left the author Kay Thomas of the submission. She has contributed to the manuscript all the way. Hence, the correct author group should be as given below:

Tomas Andri Axelsson^1^ · Cecilia Cracco^2^ · Mahesh Desai^3^ · Mudhar Nazar Hasan^1^ · Thomas Knoll^4^·· Emanuele Montanari^5^ · Daniel Perez‑Fentes^6^ · Michael Straub^7^ · Kay Thomas^8^ · James C. Williams Jr.^9^·· Marianne Brehmer^1^ · Palle J. S. Osther^10^

As a result, Authors’ contributions and Conflict of interest should be as given below:

**Authors’ contributions** TAA: Analysis of data and manuscript writing. CC, MD, TK, EM, DP-F, MS: Data collection and analysis, presentation, editing of manuscript. MH: analysis of data and editing of manuscript. KT, JCW, Jr.: analysis of data and editing of manuscript. MB: coordinator of project, analysis of data, presentation, writing and editing of manuscript. PJSO: coordinator of project, analysis of data, presentation, writing and editing of manuscript.

**Conflict of interest** TA Axelson, M Desai, MN Hasan, E Montanari, D Pérez-Fentes, M Brehmer, K Thomas, JC Williams Jr.: no conflicts of interest. C Cracco: tutor of courses for Boston Scientific; T Knoll: consultant for Storz Medical, Dornier, Olympus, and Boston Scientific; M Straub: consultant for Richard Wolf Endoscopes, Coloplast-Porgès, and Ambu; PJS Osther: consultant for Boston Scientific, Olympus, Coloplast and Bonvisi AB. None of the authors were paid to participate in the Consultation. Travel and accommodation expenses were paid by the organizers of Consultation on Kidney Stones (Lillebaelt Hospital, University Hospital of Southern Denmark and Danderyd University Hospital, Karolinska Institute).

The original article has been corrected.

